# Preservation of myocardial contractility during acute hypoxia with OMX-CV, a novel oxygen delivery biotherapeutic

**DOI:** 10.1371/journal.pbio.2005924

**Published:** 2018-10-18

**Authors:** Jason Boehme, Natacha Le Moan, Rebecca J. Kameny, Alexandra Loucks, Michael J. Johengen, Amy L. Lesneski, Wenhui Gong, Brian D. Goudy, Tina Davis, Kevin Tanaka, Andrew Davis, Youping He, Janel Long-Boyle, Vijay Ivaturi, Jogarao V. S. Gobburu, Jonathan A. Winger, Stephen P. Cary, Sanjeev A. Datar, Jeffrey R. Fineman, Ana Krtolica, Emin Maltepe

**Affiliations:** 1 Department of Pediatrics, University of California, San Francisco, San Francisco, California, United States of America; 2 Omniox, Inc., San Carlos, California, United States of America; 3 Department of Clinical Pharmacology, University of California, San Francisco, San Francisco, California, United States of America; 4 Initiative for Pediatric Drug and Device Development (iPD3), San Francisco, California, United States of America; 5 School of Pharmacy, University of Maryland, Baltimore, United States of America; University of Pittsburgh, United States of America

## Abstract

The heart exhibits the highest basal oxygen (O_2_) consumption per tissue mass of any organ in the body and is uniquely dependent on aerobic metabolism to sustain contractile function. During acute hypoxic states, the body responds with a compensatory increase in cardiac output that further increases myocardial O_2_ demand, predisposing the heart to ischemic stress and myocardial dysfunction. Here, we test the utility of a novel engineered protein derived from the heme-based nitric oxide (NO)/oxygen (H-NOX) family of bacterial proteins as an O_2_ delivery biotherapeutic (Omniox-cardiovascular [OMX-CV]) for the hypoxic myocardium. Because of their unique binding characteristics, H-NOX–based variants effectively deliver O_2_ to hypoxic tissues, but not those at physiologic O_2_ tension. Additionally, H-NOX–based variants exhibit tunable binding that is specific for O_2_ with subphysiologic reactivity towards NO, circumventing a significant toxicity exhibited by hemoglobin (Hb)-based O_2_ carriers (HBOCs). Juvenile lambs were sedated, mechanically ventilated, and instrumented to measure cardiovascular parameters. Biventricular admittance catheters were inserted to perform pressure-volume (PV) analyses. Systemic hypoxia was induced by ventilation with 10% O_2_. Following 15 minutes of hypoxia, the lambs were treated with OMX-CV (200 mg/kg IV) or vehicle. Acute hypoxia induced significant increases in heart rate (HR), pulmonary blood flow (PBF), and pulmonary vascular resistance (PVR) (*p* < 0.05). At 1 hour, vehicle-treated lambs exhibited severe hypoxia and a significant decrease in biventricular contractile function. However, in OMX-CV–treated animals, myocardial oxygenation was improved without negatively impacting systemic or PVR, and both right ventricle (RV) and left ventricle (LV) contractile function were maintained at pre-hypoxic baseline levels. These data suggest that OMX-CV is a promising and safe O_2_ delivery biotherapeutic for the preservation of myocardial contractility in the setting of acute hypoxia.

## Author summary

While hemoglobin is the primary oxygen delivery molecule used to maintain tissue oxygenation in metazoans, many organisms have other heme-containing proteins that can bind oxygen and other diatomic gases. Here, we tested whether a member of the H-NOX family of heme-containing proteins found in the thermostable bacterium *Thermoanaerobacter tengcongensis* can be engineered to deliver oxygen to severely hypoxic tissues in large mammals. This class of molecules has the advantage of high oxygen affinity and minimal nitric oxide reactivity. We demonstrate that these molecules can effectively deliver oxygen to a lamb heart with induced severe hypoxia, without overexposing the animal to oxygen or triggering systemic vascular reactivity. These molecules thus represent a novel class of oxygen delivery biotherapeutics to specifically target hypoxic tissue beds without the toxicity concerns of hemoglobin-based oxygen carriers. As tissue hypoxia is a central feature of many disease processes, this therapeutic approach may have broad clinical applicability.

## Introduction

Inadequate oxygen (O_2_) delivery relative to metabolic demand leads to progressive bioenergetic collapse and cellular dysfunction. When systemic, this defines the clinical entity of shock, a major cause of morbidity and mortality in both adults and children [1,2,3,4]. Rather than a specific disease state, shock is a shared pathologic end point arising from disorders such as respiratory failure, hemorrhage, or sepsis that ultimately impair cardiovascular function. For this reason, maintaining a balance between myocardial O_2_ supply and demand underlies a central therapeutic framework of critical care medicine.

Of all organs, the heart is metabolically unique both in regard to its energetic demands as well as its O_2_ utilization and extraction characteristics. Given its primary physiologic function as a continuous generator of mechanical force, the heart requires an extraordinary supply of biochemical energy and exhibits a far greater rate of ATP turnover than any other organ [5]. Furthermore, the heart is exquisitely dependent on aerobic metabolism to meet these high bioenergetic needs, without the ability to derive any meaningful contribution from anaerobic pathways such as glycolysis [6]. This is reflected in the large myocardial volume devoted to mitochondria and the heart’s status as the highest O_2_ consumer per gram tissue mass of any organ [5,6]. Importantly, its high O_2_ extraction ratio results in lower venous O_2_ contents than other tissues, with a significant fraction of cardiomyocytes being exposed to physiologically hypoxic environments at baseline [7,8]. When myocardial O_2_ supply becomes limited in the face of increased demand, dramatic increases in coronary blood flow as well as cardiomyocyte O_2_ extraction attempt to compensate [7,8,9]. When inadequate, biochemical signs of a switch to anaerobic metabolism are accompanied by an immediate impairment of contractile function [10]. O_2_ consumption is thus vital to provide the biochemical energy required to maintain cardiac mechanical function.

**Fig 1:**
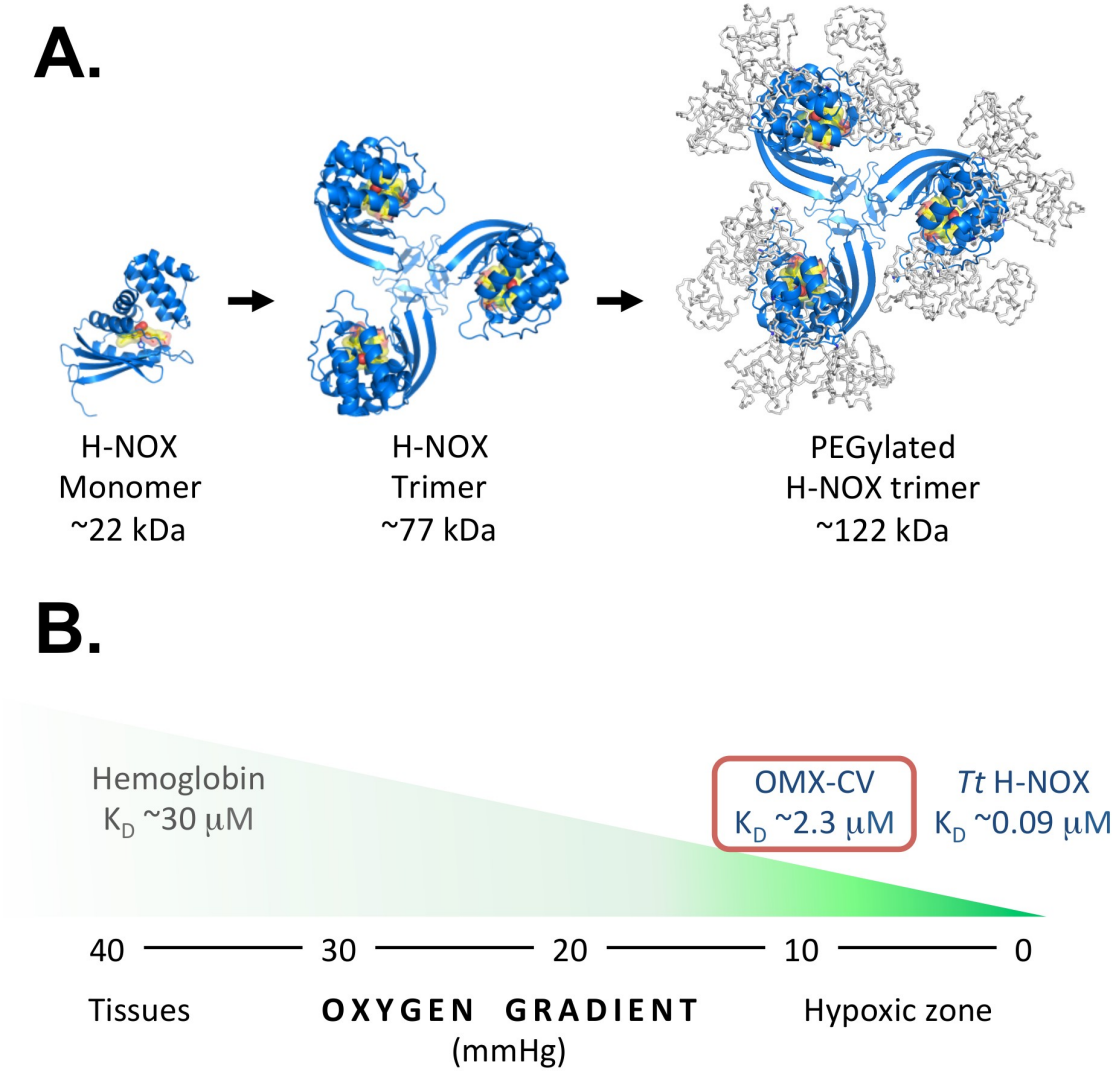
Illustration of OMX-CV H-NOX protein and its oxygen-binding characteristics. (A) Ribbon diagrams depicting an H-NOX protein monomer, H-NOX protein trimer, and PEGylated H-NOX protein trimer. The heme cofactor and the bound oxygen are depicted in yellow and red. Models were made using a Tt H-NOX structure (PDB ID 1U4H) and PyMOL [17]. (B) Illustration depicting the relative oxygen affinities of hemoglobin, *Tt* H-NOX, and OMX-CV overlaid on an oxygen gradient from normoxia to hypoxia. The oxygen affinity of hemoglobin facilitates release of oxygen in peripheral tissues (PO_2_ of about 40 mmHg), while the oxygen affinity of OMX-CV facilitates release of oxygen into hypoxic tissues (PO_2_ of about 10 mmHg). **H-NOX**, heme-based nitric oxide/oxygen; **K**_**D**_, **dissociation constant; mmHg, millimeters mercury; OMX-CV**, Omniox-cardiovascular; PEG, polyethylene glycol; PO_2_, partial pressure of oxygen; Tt, *Thermoanaerobacter tengcongensis*.

In this study, we describe, for the first time, the use of a novel O_2_ delivery biotherapeutic to alleviate hypoxia-induced tissue dysfunction in the heart. Derived from the heme-based nitric oxide (NO)/oxygen (H-NOX) sensing proteins found in the thermostable bacterium *Thermoanaerobacter tengcongensis* (Tt) [11], the protein component of Omniox-cardiovascular (OMX-CV) is engineered via trimerization and polyethylene glycol (PEG)-ylation (as illustrated in Fig 1A) to increase circulation half-life, and alterations to the heme-binding pocket to fine-tune both selectivity and avidity of interaction with the diatomic gases NO and molecular O_2_ [12,13]. Unlike hemoglobin (Hb)-based O_2_ delivery biotherapeutics that scavenged NO and therefore triggered significant vascular sequelae, including hypertension, renal dysfunction, and increased risk of myocardial infarction and death [14,15,16], the protein component of OMX-CV is uniquely tuned to bind molecular O_2_ in a way that reduces NO reactivity 50-fold compared with Hb [13], alleviating the potential risk of vasoconstriction.

Additionally, relative to Hb, the protein component of OMX-CV binds to O_2_ with a very high affinity, exhibiting a dissociation constant (K_D_) of about 2.4 μM [13]. Fig 1B shows a schematic comparing the O_2_ affinities of wild-type Tt H-NOX and OMX-CV with that of Hb, and illustrates how OMX-CV can effectively deliver O_2_ only to tissues that are significantly hypoxic while bypassing those at physiologic O_2_ tensions. Following O_2_ delivery within the hypoxic capillary environment, the unbound OMX-CV molecules enter the systemic venous and pulmonary vascular beds. In this manner, OMX-CV circulates and can be predicted to sustain an ongoing, targeted O_2_ delivery to the most hypoxic organs and tissues without unnecessary and potentially harmful [18] oxygenation of tissues at physiologic O_2_ tensions.

We hypothesized that in the setting of severe myocardial hypoxia, OMX-CV administration would increase O_2_ delivery to the heart and improve cardiac mechanical function. In order to test this hypothesis, we utilized a juvenile lamb model of severe acute alveolar hypoxia. The lamb is a robust large animal model that has been extensively utilized because of its close approximation of human cardiovascular function [19]. Here, we present data regarding the safety and efficacy of OMX-CV administration in the setting of systemic hypoxia supporting the use of OMX-CV as a promising novel O_2_ delivery biotherapeutic.

## Results

### Acute alveolar hypoxia induces a dramatic physiologic response

**Fig 2:**
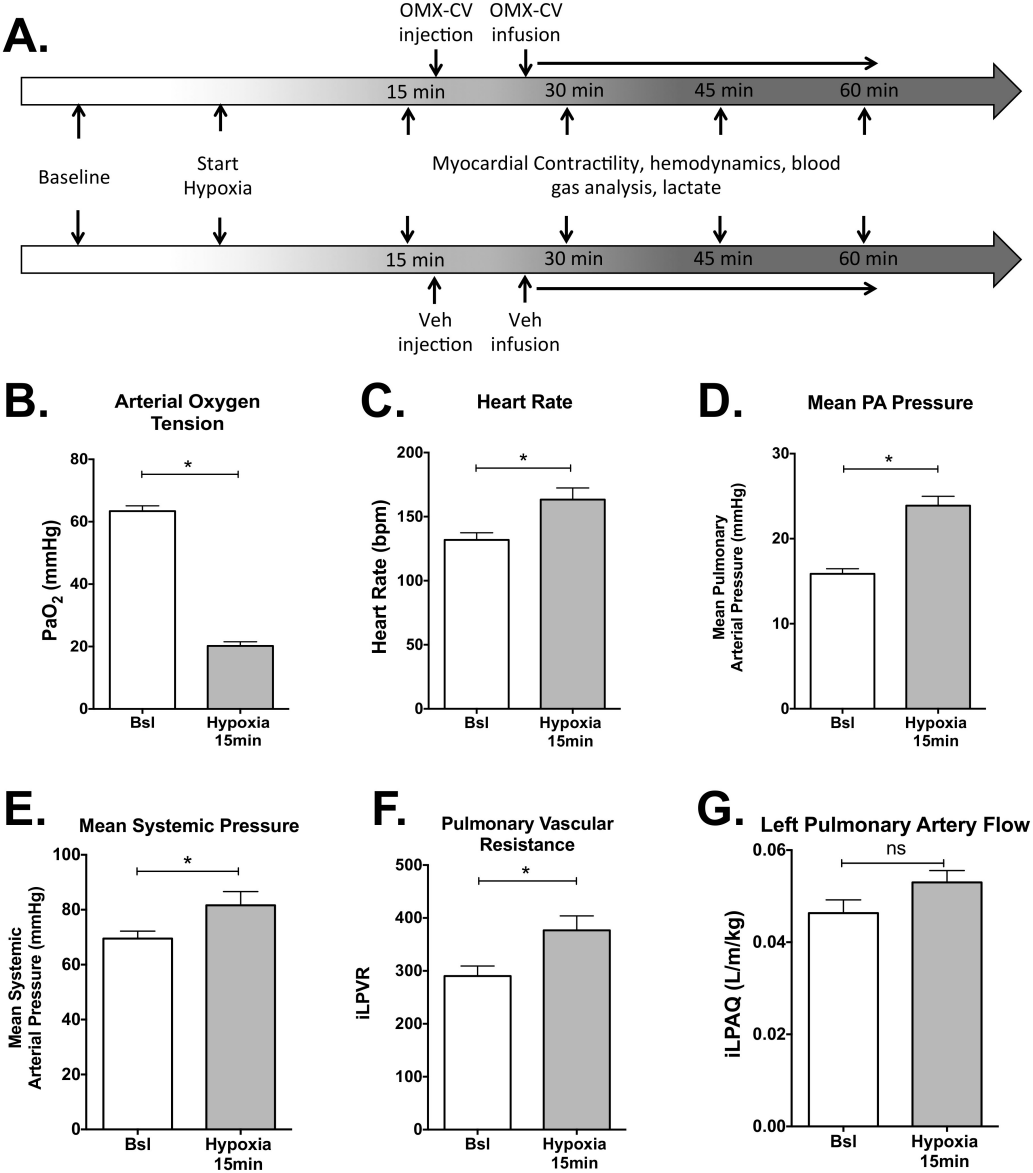
Physiologic responses of the cardiovascular system to acute alveolar hypoxia. (A) Schematic of experimental protocol. Physiologic measurements were continuously recorded and logged every second for the duration of the study. At each designated time point, physiologic data were averaged over a 60-second period in 5-second intervals. (B) Average measured PaO_2_ in mmHg of all animals (*n* = 13) at baseline (Bsl) compared with 15 minutes following institution of hypoxic ventilation. (C) Average heart rate of all animals at Bsl compared with 15 minutes following institution of hypoxic ventilation. (D) Average mean pulmonary arterial pressure (in mmHg) of all animals at Bsl compared with 15 minutes following institution of hypoxic ventilation. (E) Average mean systemic arterial pressure (in mmHg) of all animals at Bsl compared with 15 minutes following institution of hypoxic ventilation. (F) Average indexed PVR of all animals at baseline (Bsl) compared with 15 minutes following institution of hypoxic ventilation. PVR of the left lung was calculated as the difference of mean pulmonary arterial pressure and left atrial pressure divided by the indexed LPA blood flow. (G) Average indexed left pulmonary arterial blood flow of all animals at Bsl compared with 15 minutes following institution of hypoxic ventilation. Flow was indexed to body size by dividing by the animal’s weight in kilograms. In all figures, “*” denotes significance with *p* < 0.05, while “ns” denotes *p* > 0.05. Error bars demonstrate standard error of the mean. Primary data can be found in S1 Table. bpm, beats per minute; Bsl, baseline; iLPAQ, indexed left pulmonary artery flow; iLPVR, indexed left pulmonary vascular resistance; LPA, left pulmonary artery; mmHg, millimeters mercury; OMX-CV, Omniox-cardiovascular; PA, pulmonary artery; PaO_2_, arterial oxygen tension; Veh, vehicle.

**Fig 3:**
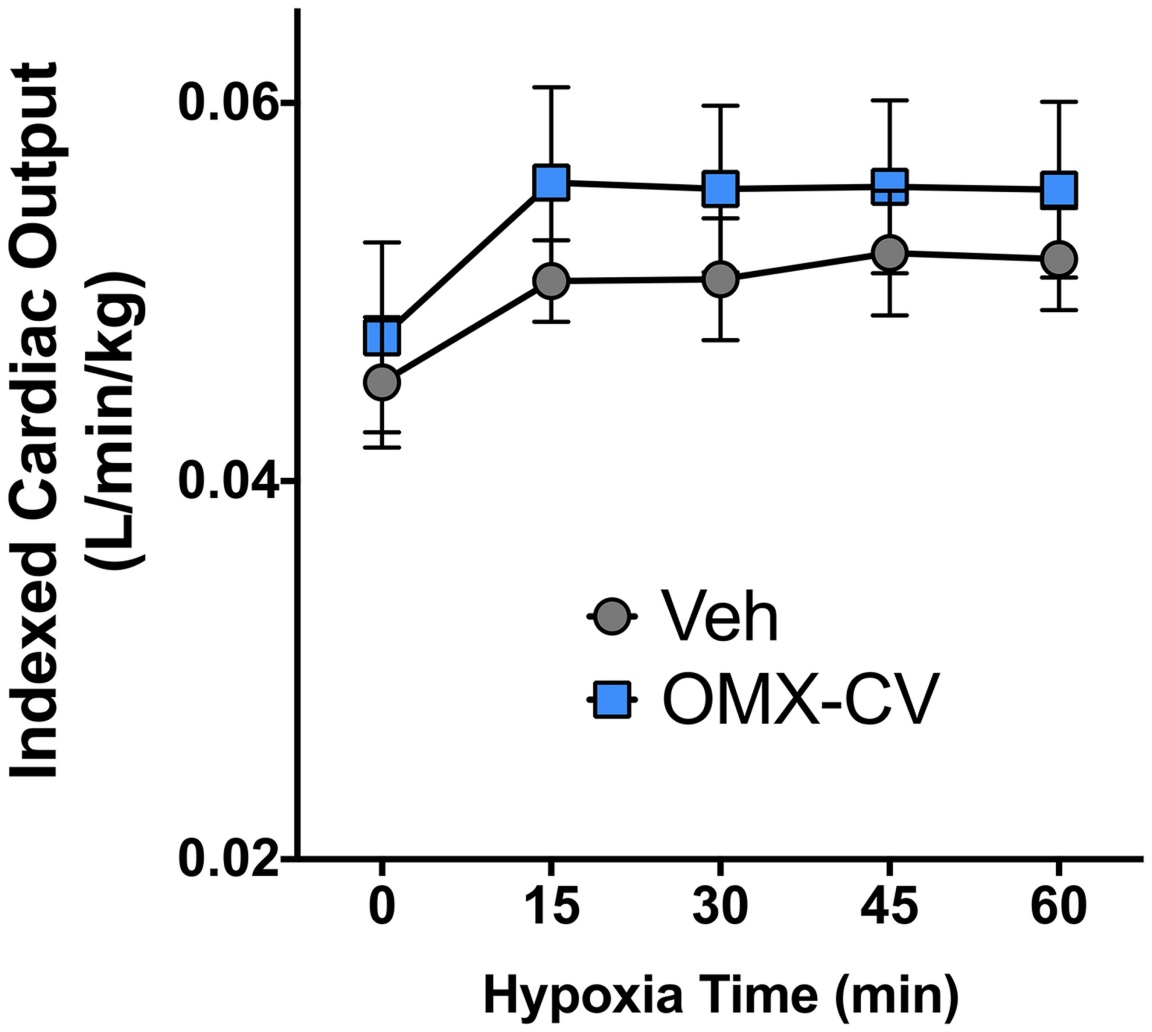
Cardiac output in control and OMX-CV–treated animals. Indexed left pulmonary arterial blood flow in vehicle- versus OMX-CV–treated groups over the duration of the experimental protocol. Time 0 represents the physiologic baseline and other time points represent total duration of hypoxic ventilation. Error bars correspond to the standard error of the mean. There is a statistically significant interaction between time and iLPA flow (*p* <0.05) by two-way ANOVA. There is no significant difference between OMX-CV (*n* = 6) and vehicle (*n* = 7) groups. Primary data can be found in S1 Table. iLPA, indexed left pulmonary artery; OMX-CV, Omniox-cardiovascular; Veh, vehicle.

Previous studies have described the acute cardiovascular response to progressive alveolar hypoxia in large animal models [20,21]. Here, we established a model of acute alveolar hypoxia in juvenile lambs triggered via inhalation of a gas mixture containing 10% O_2_ (Fig 2A). Physiologic data were compared at pre-hypoxic baseline and at 15 minutes of hypoxia (prior to experimental intervention) for all animals included in the analysis (*n* = 13) As expected, we witnessed a precipitous fall in arterial O_2_ tension (PaO_2_) with the onset of alveolar hypoxia that was then sustained for the duration of the study (Fig 2B). Fig 2C–2F demonstrate the dramatic changes in physiologic parameters that accompany this severe hypoxemia at the 15-minute time point. The animals all exhibit acute increases in heart rate (HR), systemic blood pressure (systolic and mean), pulmonary blood pressure (systolic, diastolic, and mean), and left and right atrial pressures. As expected [22], there is a significant increase in pulmonary vascular resistance (PVR) attributable to hypoxic pulmonary vasoconstriction. However, there is not a significant alteration in either systemic diastolic blood pressure or systemic vascular resistance (SVR). Additionally, there is an overall increase in cardiac output of approximately 15% (Fig 2G). Although this just fails to reach statistical significance when evaluated at the 15-minute time point (*p* = .063), there is a significant increase in cardiac output amongst all animals (but no between-group difference) when evaluated over the duration of the hypoxic exposure (Fig 3). Table 1 provides additional cardiovascular physiologic parameters comparing OMX-CV and vehicle groups at their respective hypoxic baselines (before drug) and study conclusion (60 minutes).

**Table 1:** A compilation of cardiovascular physiologic parameters measured during hypoxic conditions in lambs receiving vehicle or OMX-CV. Primary data can be found in S1 Table.

Parameters	Time point	Vehicle	OMX-CV	*p*-value
		Avg ± SD	Avg ± SD	
Hgb		9.38 ± 1.2	9.32 ± 1.5	0.83
PaO_2_	Hypoxia Bsln	18 ± 2.3	21 ± 4.3	0.28
	Hypoxia 60 min	22.7 ± 1.7	21.7 ± 2.1	0.78
Systolic SAP	Hypoxia Bsln	127.9 ± 24	119.3 ± 28.5	0.65
	Hypoxia 60 min	105.5 ± 23.9	102.43 ± 29.7	0.77
Diastolic SAP	Hypoxia Bsln	64.1 ± 19.7	60.4 ± 18.7	0.98
	Hypoxia 60 min	42.0 ± 10.5	48.9 ± 19.8	0.55
Mean SAP	Hypoxia Bsln	81.9 ± 19.9	81.3 ± 17.3	0.83
	Hypoxia 60 min	61.3 ± 14	67.7 ± 19.1	0.60
HR	Hypoxia Bsln	158.0 ± 19.5	169.6 ± 44.9	0.50
	Hypoxia 60 min	193.4 ± 28.2	184.1 ± 24.3	0.75
Systolic PAP	Hypoxia Bsln	35.3 ± 4.0	38.0 ± 9.4	0.57
	Hypoxia 60 min	36.0 ± 3.3	39.5 ± 8.2	0.38
Diastolic PAP	Hypoxia Bsln	15.4 ± 3.9	13.5 ± 3.8	0.46
	Hypoxia 60 min	16.2 ± 2.3	15.7 ± 3.8	0.92
Mean PAP	Hypoxia Bsln	24.0 ± 3.5	23.7 ± 4.8	0.81
	Hypoxia 60 min	25.0 ± 2.3	25.9 ± 4.7	0.68
LAP	Hypoxia Bsln	3.5 ± 1.9	5.7 ± 1.9	0.07
	Hypoxia 60 min	4.4 ± 1.7	5.7 ± 1.6	0.02
RAP	Hypoxia Bsln	2.8 ± 1.2	4.6 ± 1.5	0.05
	Hypoxia 60 min	4.8 ± 2.5	4.9 ± 1.8	0.94
iLPAQ	Hypoxia Bsln	0.051 ± 0.006	0.056 ± 0.012	0.36
	Hypoxia 60 min	0.052 ± 0.007	0.055 ± 0.011	0.56
iLPAVR	Hypoxia Bsln	414.9 ± 107.1	332.2 ± 70.1	0.12
	Hypoxia 60 min	402.3 ± 62.3	375.3 ± 97.9	0.46

Abbreviations: Avg, average; Bsln, baseline; Hgb, hemoglobin; HR, heart rate; iLPAQ, indexed left pulmonary artery flow; iLPAVR, indexed left pulmonary artery vascular resistance; LAP, left atrial pressure; OMX-CV, Omniox-cardiovascular; PaO_2_, arterial oxygen tension; PAP, pulmonary artery pressure; RAP, right atrial pressure; SAP, systemic arterial pressure.

### OMX-CV administration does not cause systemic or pulmonary vasoconstriction

**Fig 4:**
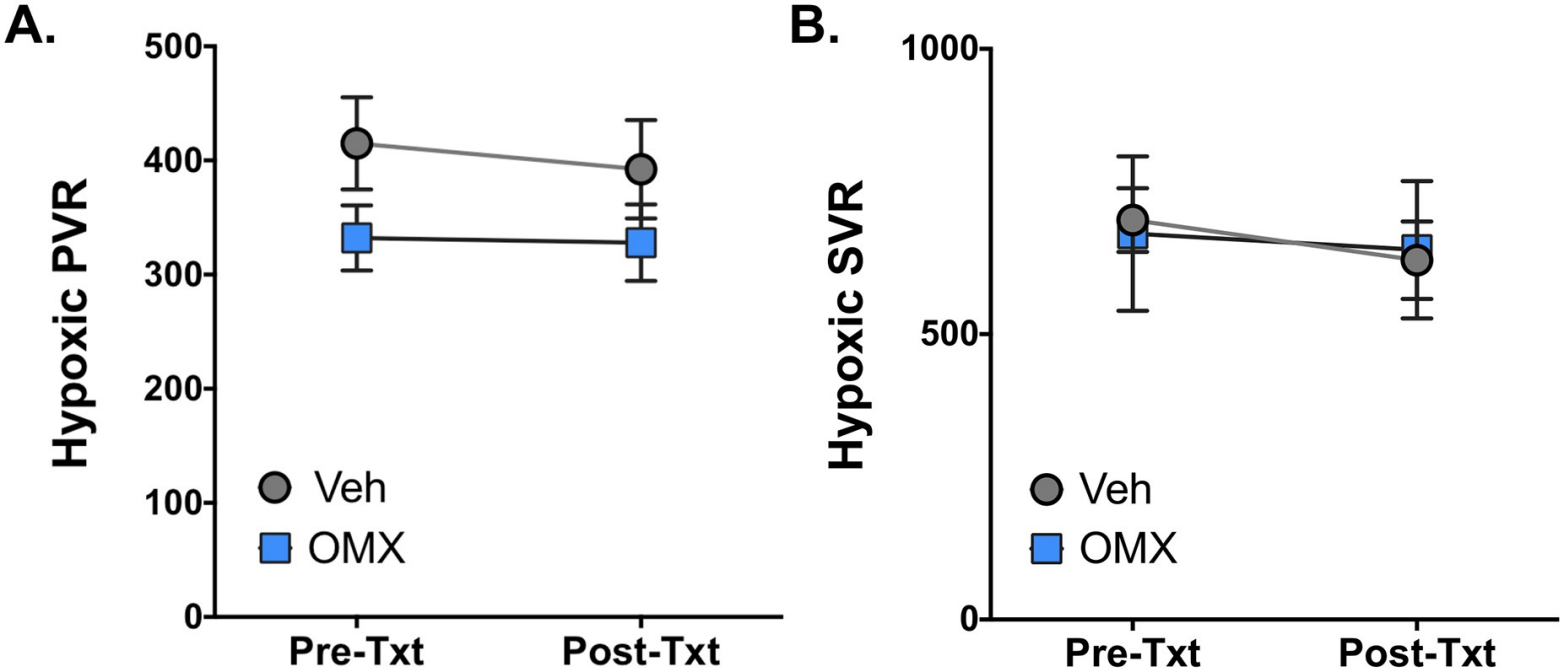
SVR and PVR before and after OMX-CV and vehicle administration. (A) Indexed PVR in vehicle- (*n* = 7) and OMX-CV–treated (*n* = 6) animals during hypoxic ventilation immediately prior to (pre-txt) and following (post-txt) treatment administration. There are no statistically significant differences between groups or within groups pre- and posttreatment. Error bars represent the standard error of the mean. (B) Indexed SVR in vehicle- and OMX-CV–treated animals pre-txt and post-txt. There are no statistically significant differences between groups or within groups pre- and posttreatment. Error bars represent the standard error of the mean. Primary data can be found in S1 Table. OMX-CV, Omniox-cardiovascular; post-txt, immediately following treatment administration; pre-txt, immediately prior to treatment administration; PVR, pulmonary vascular resistance; SVR, systemic vascular resistance; Veh, vehicle.

Taking into consideration the historical challenges related to NO scavenging encountered in the use of hemoglobin-based oxygen carriers (HBOCs), we evaluated the physiologic impact of OMX-CV administration on systemic and pulmonary vascular reactivity. Importantly, the total amount of OMX-CV administered relative to circulating Hb is quite low. In an average 10-kg lamb with a serum Hb concentration of 10 g/dL and a circulating blood volume of 70 mL/kg, Hb O_2_ carrying capacity is approximately 4.8 mM. For these experiments, we provided approximately 54 mL total of OMX-CV infusion, representing an infused OMX-CV O_2_-binding capacity of approximately 0.1 mM, or 2% that of circulating Hb. As noted in Table 1, this does not result in appreciable differences in circulating PaO_2_ values but is readily available for oxygenating severely hypoxic tissues. Given the substantial physiologic changes induced by the hypoxic stimulus, we specifically evaluated effects on SVR and PVR in the setting of systemic hypoxia prior to and immediately following drug or vehicle administration (*n* = 7 control and *n* = 6 OMX-CV). As seen in Fig 4, we observed no significant increase in either the indexed PVR (Fig 4A) or indexed SVR (Fig 4B) with administration of OMX-CV when compared with vehicle control under hypoxic conditions. Furthermore, there was no difference in the absolute value or percent change between the OMX-CV–treated and vehicle-treated groups. While hypoxia clearly results in a pre-constricted pulmonary vasculature, this occurs through a NO-independent mechanism, and PVR would be expected to remain quite sensitive to abrupt changes in NO signaling [23,24]. Additionally, SVR is also increased during hypoxia, as evidenced by increased mean systemic pressure, and was similarly unaffected by OMX-CV administration (Fig 4B), affirming a lack of direct vasoreactivity.

### OMX-CV decreases myocardial hypoxia

**Fig 5:**
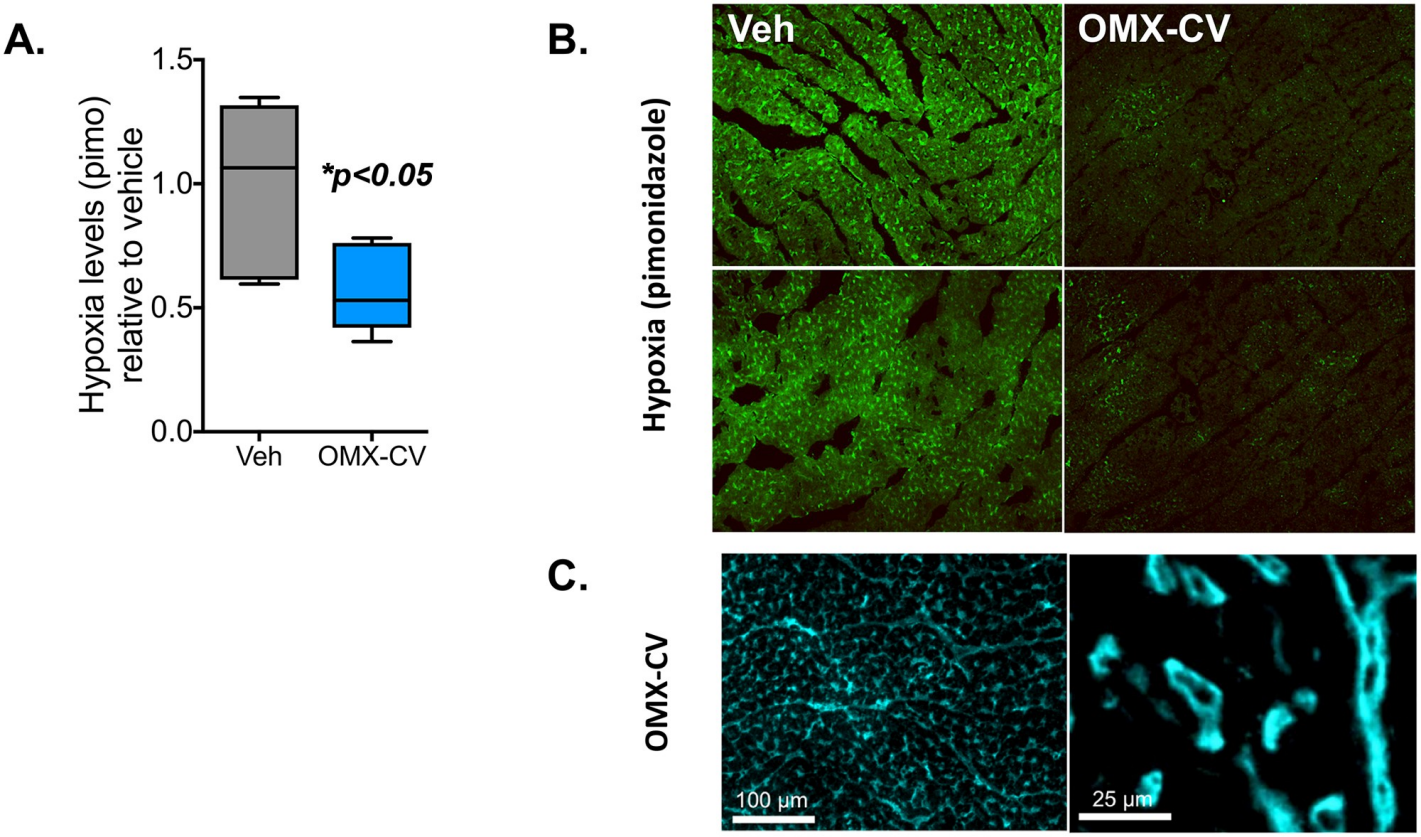
Myocardial hypoxia in control and OMX-CV–treated animals. In a subset of vehicle- and OMX-CV–treated animals (*n* = 3 each), following measurement of physiologic parameters, pimonidazole was administered intravenously and tissues were collected for analysis 30 minutes later. (A) Quantification of pimonidazole adducts in vehicle- and OMX-CV–treated myocardial tissue by pimonidazole ELISA. Values are ±SEM, *p < 0.05 by Student *t* test. (B) Representative images of vehicle- and OMX-CV–treated myocardium tissue sections immunostained with antibodies targeting pimonidazole adducts. (C) Representative images of OMX-CV–treated myocardial tissue sections immunostained with antibodies targeting the OMX-CV molecule. OMX-CV, Omniox-cardiovascular; pimo, pimonidazole; Veh, vehicle.

To directly assess the effect of OMX-CV on myocardial tissue oxygenation, following the final assessment of physiologic parameters, pimonidazole (Hypoxyprobe, 85 mg/kg), a well-established marker of tissue hypoxia [25], was administered intravenously to a subset of animals (*n* = 3 per treatment group). Thirty minutes after administration of pimonidazole, the animals were humanely killed and tissues collected for processing and measurement of pimonidazole adduct levels in the ventricular myocardium. Pimonidazole freely diffuses into cells and is competitively metabolized via oxidative or reductive chemical reactions, depending on the tissue O_2_ content. In severely hypoxic environments (below 10 mm Hg), reductive metabolism is favored and in its reduced state, pimonidazole forms covalent adducts with sulfhydryl groups of proteins and glutathione, leading to accumulation of pimonidazole adducts inside the cell [25]. Pimonidazole adducts can be recognized using pimonidazole-targeted primary antibodies and quantified using standard ELISA and immunofluorescent (IF) methods. As seen in Fig 5A & 5B, the OMX-CV–treated animals exhibited a significant reduction in myocardial hypoxia compared with controls, as evidenced by lower levels of bound pimonidazole observed via IF microscopy and quantified by ELISA. To verify that the improved myocardial tissue oxygenation in the OMX-CV group was mediated by transcapillary O_2_ diffusion, rather than vascular extravasation, IF microcopy was performed with antibodies directed against OMX-CV. As seen in Fig 5C, OMX-CV localized within the capillary vascular spaces throughout the heart and not the extracellular spaces surrounding the cardiomyocytes. Thus, at tested doses, a high-affinity O_2_ delivery biotherapeutic can relieve tissue hypoxia in the heart.

### OMX-CV preserves myocardial contractility during systemic hypoxia

To determine whether this improvement in myocardial O_2_ delivery translates into a physiologic benefit, we utilized cardiac pressure volume loop analysis to evaluate contractile function of the bilateral ventricles. As noted previously by other groups, evaluation of cardiac function in intact animal studies is often obscured by compensatory physiologic alterations to ventricular loading conditions and sympathetic tone [10,21]. Indeed, we observed in our own data that from the onset of hypoxia, both the OMX-CV and control groups exhibited similar elevations in cardiac output (about 15%) above the normoxic baseline, and that this was sustained throughout our study (Fig 3). This suggests a full mobilization of compensatory mechanisms that may account for the lack of a significant difference in cardiac output between the OMX-CV and control groups at early time points. Initially advanced by Suga and Sagawa in the 1970s [26], evaluation of two-dimensional ventricular pressure-volume (PV) loops with a focus on the end systolic pressure-volume relationship (ESPVR) is now the widely adopted standard used to assess the load-independent contractile state of the ventricles [27]. This method has previously been used to validate the hypoxic depression of myocardial contractile function in dogs and shown to correlate closely with myocardial O_2_ deficiency and the onset of anaerobic metabolism [8,10].

**Fig 6:**
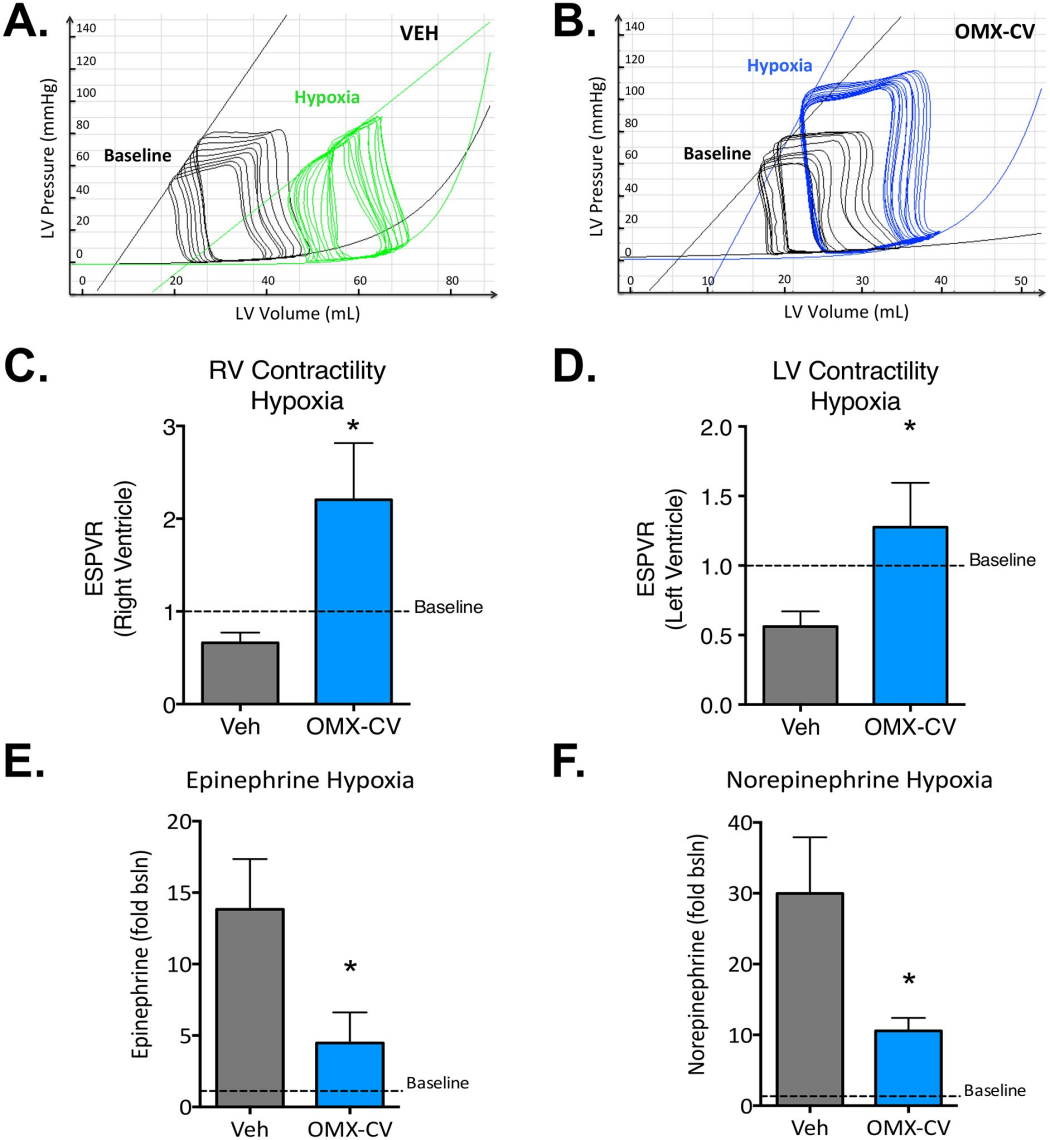
Ventricular contractility and circulating catecholamine levels in control and OMX-CV–treated animals. (A) Representative Pressure-Volume loops obtained from the left ventricle of a vehicle-treated animal during transient IVC occlusion. LV pressure is measured on the y-axis and LV volume on the x-axis. The superimposed line tangential to the end systolic pressure volume points of each family of loops defines the ESPVR. The family of loops in black and their corresponding ESPVR were obtained during the physiologic baseline, while the green loops and ESPVR were obtained from the same animal following 1 hour of hypoxic ventilation. (B) Representative Pressure-Volume loops obtained from the LV of an OMX-CV–treated animal during transient IVC occlusion. The family of loops in black and their corresponding ESPVR were obtained during the physiologic baseline, while the blue loops and ESPVR were obtained from the same animal following 1 hour of hypoxic ventilation. (C) Mean right ventricular contractility (as assessed by slope of the ESPVR relative to baseline) in vehicle- (*n* = 7) and OMX-CV–treated (*n* = 6) animals after 1 hour of hypoxic ventilation. Error bars represent the standard error of the mean; “*” denotes a significant difference between groups with *p* < 0.05. (D) Mean left ventricular contractility (as assessed by slope of the ESPVR relative to baseline) in vehicle- (*n* = 7) and OMX-CV–treated (*n* = 6) animals after 1 hour of hypoxic ventilation. Error bars represent the standard error of the mean; “*” denotes a significant difference between groups with *p* < 0.05. (E) Mean serum epinephrine levels (expressed as fold change relative to physiologic baseline) after 1 hour of hypoxic ventilation in vehicle- (*n* = 7) and OMX-CV–treated (*n* = 6) animals. Error bars represent the standard error of the mean; “*” denotes a significant difference between groups with *p* < 0.05. (F) Mean serum norepinephrine levels (expressed as fold change relative to physiologic baseline) at 1 hour of hypoxic ventilation in vehicle- and OMX-CV–treated animals. Error bars represent the standard error of the mean; “*” denotes a significant difference between groups with *p* < 0.05. Primary data can be found in S1 Table. bsln, baseline; ESPVR, end systolic pressure-volume relationship; IVC, inferior vena cava; LV, left ventricle; mmHg, millimeters mercury; OMX-CV, Omniox-cardiovascular; RV, right ventricle; Veh, vehicle.

In order to delineate the ESPVR, a family of loops was generated (as seen in Fig 6A & 6B) through transient preload suppression induced by graduated occlusion of the inferior vena cava (IVC). The slope of the tangent connecting the end systolic points of these loops gives the most precise representation of intrinsic contractility of the ventricle. As seen in Fig 6A, which shows a representative set of loops and their ESPVR from the LV of a control animal, the decline in slope from baseline (black) to hypoxia (green) demonstrates a decrease in contractility. In contrast, the LV loops of an OMX-CV–treated animal (Fig 6B) exhibit an increasing slope, indicating an improvement in contractile function. By normalizing the slope of the ESPVR at 60 minutes to the baseline for each animal (*n* = 7 control and *n* = 6 OMX-CV), we observed that OMX-CV–treated animals maintained an average contractility up to 2-fold above their own baseline under hypoxic conditions (Fig 6C), while RV (Fig 6C) and LV (Fig 6D) contractility were both reduced in vehicle controls. These data indicate that OMX-CV treatment was able to reverse the effects of myocardial hypoxia and preserve cardiac contractility.

We finally explored the role of sympathetic activation in the cardiovascular response to acute alveolar hypoxia by measuring plasma levels of the sympathetic hormones epinephrine and norepinephrine at baseline and after 60 minutes of hypoxia. Released by the adrenal medulla in response to increased stimulation of the sympathetic nervous system, these hormones exhibit potent cardiovascular effects mediated through binding of alpha- and beta-adrenergic receptors in the heart and vasculature. Similar to what has been described [21], we noted a significant increase in the levels of these catecholamines under hypoxic stress, marking an activated sympathetic response. Interestingly, we found a significant difference in the levels of epinephrine and norepinephrine between the OMX-CV– and vehicle-treated animals (*n* = 7 control and *n* = 6 OMX-CV), with hypoxia inducing an approximately 3-fold higher increase in both hormones in the vehicle group compared with OMX-CV (Fig 6E and 6F). Thus, increased adrenergic signaling was not responsible for the improved myocardial contractility of OMX-CV–treated animals compared with the control group, although the improved performance in the presence of the lower induction of catecholamines suggests a greater capacity of the OMX-CV–treated myocardium to respond to adrenergic signaling under hypoxic stress. We therefore conclude that while cardiac output can be maintained during severe acute alveolar hypoxia through diverse adaptive mechanisms, OMX-CV directly improves the intrinsic contractile function of the heart by virtue of its ability to increase myocardial O_2_ content.

## Discussion

Here, we have provided preclinical data highlighting the therapeutic efficacy of the OMX-CV biotherapeutic in relieving hypoxic myocardial dysfunction in a large animal model. H-NOX–based variants are ideally suited for O_2_ delivery to hypoxic tissues, such as the myocardium, because of their O_2_ affinity as well as pharmacokinetic and safety profiles [13]. OMX-CV’s O_2_ affinity aligns extremely well with the unique O_2_ demands and microenvironments encountered within the stressed heart, its half-life enables long-term efficacy following single intravenous infusion, and its O_2_ specificity minimizes the vasoactive side effects encountered with HBOCs.

The cardiovascular system responds to acute hypoxia by attempting to augment and enhance systemic O_2_ delivery. Cardiac output increases with accompanying elevations in both HR and contractile state, which further escalate myocardial O_2_ demand. In response to the high and variable demand for O_2_ during states of acute stress, as well as the tight interrelationship between myocardial function and O_2_ supply, the heart has evolved robust adaptive mechanisms to augment myocardial O_2_ delivery and extraction [28]. For example, during exercise-induced elevations in cardiac output, O_2_ utilization may increase by greater than 5-fold, supported by substantial increases in coronary blood flow, capillary recruitment, and increased O_2_ extraction [7]. Even under unstressed conditions, the heart exhibits a high O_2_ extraction ratio with a correspondingly low venous saturation. When demand increases, the heart has a unique capacity to increase extraction to a greater extent than other tissues [8].

Cain and colleagues initially demonstrated that global hypoxic hypoxia and anemic hypoxia induced global anaerobic metabolism at greatly differing values of mixed venous partial pressure of oxygen (PO_2_) [9]. These differences in tissue responses to the same level of hypoxia in the blood implied that simple diffusion forces are not the limiting factor to tissue O_2_ extraction, and Schumacker and colleagues subsequently confirmed that a constant critical O_2_ extraction ratio exists in dogs [29]. Although the exact mechanisms underlying these differences are unclear, the physiologic consequence is that most tissues will start to experience O_2_ deficiency despite a relatively high average O_2_ saturation of the blood exiting their capillaries. In contrast to the other tissues and organs, the myocardium can achieve a substantially higher O_2_ extraction ratio, only exhibiting signs of anaerobic metabolism at a critically low coronary venous saturation [8]. This markedly hypoxic venous and end capillary blood reflects a correspondingly hypoxic tissue bed, creating the ideal cellular microenvironment to facilitate O_2_ dissociation and delivery by OMX-CV. Consistent with this prediction, we have shown here that in the stressed, hypoxic lamb heart, myocardial oxygenation and contractile function can be preserved with the administration of OMX-CV. This is particularly remarkable given that the total amount of OMX-CV used in our studies equates to only approximately 2% of the total O_2_ carrying capacity of the circulating Hb. Importantly, the small amount of OMX-CV administered relative to total circulating Hb serves to limit any potential negative impact on total O_2_ bioavailability.

Furthermore, the high O_2_ affinity of OMX-CV precludes O_2_ delivery under non-hypoxic conditions. This is in marked contrast to the less avid delivery profile of Hb and most HBOCs, which have been shown to contribute to pathologic hyperoxygenation of tissue and circulatory microenvironments [30]. This excessive O_2_ release has been shown to cause oxidative stress to the tissues through the production of toxic reactive oxygen species (ROS) and to induce detrimental microvascular shunting mechanisms that may inappropriately impair tissue perfusion. Delivery of excess O_2_ in the setting of shock is a frequent contributor to microcirculatory shunting with significant clinical consequences [31]. While vascular indices can frequently be normalized within the macrocirculation in the setting of shock, tissue perfusion can nevertheless be compromised because of shunting at the microcirculatory level. Importantly, in adult patients with severe sepsis and traumatic hemorrhagic shock, for example, the loss of coherence between the resuscitated macrocirculation and the microcirculation is one of the most sensitive and specific hemodynamic indicators associated with increased multi-organ failure and mortality [32,33,34,35]. Similarly, in critically ill children with sepsis, a persistently altered microcirculation has been associated with increased mortality [36]. OMX-CV allows a more targeted delivery of O_2_ to only the most hypoxic tissue beds and may help alleviate the underappreciated but significant morbidities associated with excessive tissue oxygenation in this setting.

Interestingly, we noted in our study that OMX-CV administration was associated with a smaller increase in circulating catecholamine levels in the setting of systemic hypoxia. While it is unclear what exactly underlies this difference in catecholamine production and release, it does suggest potential implications related to cardiac function. Hypoxia is a well-established stimulus for catecholamine secretion both in vitro and in vivo [37,38,39], and adrenergic responses to hypoxic stress are important for the maintenance of cardiorespiratory homeostasis [40,41]. In the perinatal period, catecholamine production by adrenomedullary chromaffin cells is directly stimulated by cellular hypoxia [42,43]. However, as mammals age, this primary cellular response to O_2_ is blunted and cholinergic innervation becomes the predominant regulatory mechanism [44]. The sympathetic response to hypoxia therefore matures to reflect the integrated input from peripheral and central chemoreceptors. In our juvenile lamb model of systemic hypoxia, OMX-CV administration appears to blunt hypoxia-driven catecholamine production. It is not clear if this reflects augmented O_2_ delivery to chemoreceptors or the chromaffin cells themselves, or perhaps represents some secondary mechanism related to more favorable hemodynamics associated with improved myocardial oxygenation. Importantly, in the control animals, diminished cardiac contractility is observed despite dramatically elevated levels of circulating catecholamines, while the OMX-CV–treated animals exhibit preserved contractility despite smaller increases in catecholamine levels. Epinephrine and norepinephrine are potent inotropes, vital to the regulation of cardiac contractility and hemodynamic function in response to physiologic stress. Here, we show that OMX-CV supports preservation of the cardiac response to these key regulators, which are important not only as endogenous hormones but also as exogenous agents heavily utilized for cardiovascular support in critical care medicine.

With respect to its safety profile, OMX-CV exhibits significant advantages over previously developed HBOCs [45]. As the protein responsible for storage and transport of O_2_ in red blood cells (RBCs) [46], Hb has been the precursor for the synthesis and formulation of HBOCs previously developed as RBC substitutes [47,48,49,50]. The first HBOC to be developed in this capacity consisted of partially purified “stroma-free” Hb [51]. However, transfusion of acellular Hb led to several major side effects [52,53,54,55,56]. Extracellular tetrameric Hb readily dissociates into two pairs of dimers [53,54], which are extremely prone to oxidation [56] and enhanced renal excretion [53,57]. Hb oxidation to methemoglobin (metHb) promotes unfolding of the globin chains and releases cytotoxic heme into the circulation, leading to kidney tubule damage and eventual renal failure [53,54]. Furthermore, metHb can no longer carry O_2_ and can also contribute to the generation of harmful ROS [52,55]. Additionally, extracellular Hb can trigger vasoconstriction and systemic hypertension by various mechanisms [30,58,59]. Foremost amongst these is the indiscriminate scavenging of NO, an important intrinsic vasodilator that is locally produced by endothelial cells to relax vascular smooth muscle [58,60]. Also, potentially important is the hyperoxygenation of local vasculature that can elicit inappropriate vasoconstriction within the microcirculation, compared to more tempered O_2_ delivery into the vessel lumen from physiologic RBC-encapsulated Hb [30,45]. Overall, the presence of extracellular Hb in the circulation may lead to direct tissue toxicity via heme release and ROS generation, while simultaneously impairing blood flow because of pathologic alterations in vasomotor tone. With its unique structure and O_2_-binding characteristics, OMX-CV averts the potential for many of these deleterious side effects. In this study, we have shown a lack of direct vasoreactivity in both the systemic and pulmonary vascular beds, providing strong evidence for selective O_2_ delivery in severely hypoxic microenvironments and lack of vasoactivity.

In summary, we present preclinical data from a large animal model highlighting the therapeutic efficacy of a novel O_2_ delivery biotherapeutic agent, OMX-CV, in relieving hypoxic myocardial dysfunction. OMX-CV is ideally suited for myocardial O_2_ delivery because of its unique O_2_-binding characteristics and safety profile. Its high O_2_ affinity complements the unique O_2_ demands and microenvironments encountered within the stressed heart, while its low reactivity with NO minimizes the vasoactive side effects encountered with HBOCs. Additionally, while exogenous O_2_ administration can increase systemic arterial O_2_ content, it can also result in microvascular shunting mechanisms that limit deep tissue oxygenation [61,62]. OMX-CV therefore has the potential to improve oxygenation in a wide range of tissues and clinical scenarios in which O_2_ delivery may be compromised.

## Materials and methods

### Ethics statement

All protocols and procedures for this work were approved by the Institutional Animal Care and Use Committee of the University of California, San Francisco. AN155428.

### Surgeries

In this study, 13 juvenile lambs (4–6 weeks of age) were anesthetized with fentanyl, ketamine, and diazepam and paralyzed with vecuronium to facilitate intubation and mechanical ventilation. Ongoing sedation and neuromuscular blockade were administered as a continuous infusion of ketamine, fentanyl, diazepam, and vecuronium. The sedative mixture was titrated to maintain age-appropriate HR. Femoral venous and arterial access were obtained via cutdown of the hind limbs, and arterial pressure was continuously transduced and recorded. The animals were ventilated with 21% FiO_2_ initially, with a positive end expiratory pressure of 5 cm H_2_O, tidal volumes of 10 mL/kg, and respiratory rate titrated to maintain pCO_2_ of 35–45 millimeters mercury (mmHg) by arterial blood gas measurements. Thoracotomy was performed and Sorenson Neonatal Transducers (Abbott Critical Care Systems, N. Chicago, IL) were introduced into the left and right atria and main pulmonary artery (MPA) to continually transduce and record pressures. An ultrasonic flow probe (Transonics Sytems, Ithaca, NY) was placed on the left pulmonary artery (LPA) to continuously monitor and record blood flow. Admittance PV catheters (Transonics Systems, Ithaca, NY) were introduced into the RV and LV via ventriculostomy to perform ventricular pressure volume analysis. These catheters consist of a solid-state sensor that directly measures pressure with high precision and excitation and recording electrodes that measure volume based on electrical admittance. Alternating current applied to the excitation electrodes generates an electrical field within the ventricle and the recording electrodes measure voltage changes, allowing calculation of resistance and conductance. With input of a measured blood resistivity and baseline stroke volume (as assessed by total cardiac output estimate from LPA flow/HR), time varying conductance can be used to solve for ventricular blood volume in real time [63]. Animals with Hb levels of less than 7.5 g/dL following surgical instrumentation were transfused with fresh whole maternal blood in increments of 5 mL/kg to achieve this minimum threshold.

Following instrumentation, the animals were allowed to recover to steady state until they required no further adjustment to sedatives and exhibited stable hemodynamic parameters. This time was designated as the normoxic baseline and blood gas analysis was performed. Baseline ventricular ESPVR was assessed by transient IVC occlusion. Following baseline assessment, the animals were subjected to sustained alveolar hypoxia by ventilation with an admixture of atmospheric gas and nitrogen to achieve a FiO_2_ of 10%. Arterial blood gas analysis was performed every 15 minutes with blood withdrawn from the femoral artery and analyzed using a Radiometer ABL5 pH/blood gas analyzer (Radiometer, Copenhagen, Denmark). Ventilatory rate was adjusted to maintain PCO_2_ 35–45 mmHg and metabolic acidosis was corrected with NaHCO_3_ boluses to maintain pH >7.30.

### Animal care and use

All protocols and procedures for this work were approved by the Institutional Animal Care and Use Committee of the University of California, San Francisco. Animals’ vital signs, including core temperature, were monitored throughout the study, and they were given intravenous fluids and prophylactic antibiotics per protocol. At the end of each protocol, all lambs were euthanized with a lethal injection of sodium pentobarbital followed by bilateral thoracotomy, as described in the NIH Guidelines for the Care and Use of Laboratory Animals.

### OMX-CV production

The engineered Tt H-NOX protein described in this study was produced by QuikChange Site-Directed Mutagenesis (Agilent), subcloned into an expression plasmid, transformed into *Escherichia coli*, and expressed essentially as described [11]. Cells were harvested by hollow-fiber tangential-flow filtration and processed immediately. The His-tagged Tt H-NOX protein was purified from cell lysate using Ni-affinity chromatography and further polished by passage over an anion-exchange column to remove remaining host cell DNA, host cell proteins, and endotoxins. The purified protein was formulated to produce OMX-CV, and frozen at −80 °C until use. Protein concentrations were determined using UV-Vis spectrophotometry as described [11]. Prior to use in animal studies, OMX-CV was subjected to purity testing by SDS-PAGE (Invitrogen) and SEC-HPLC (Agilent) and safety testing by kinetic chromogenic LAL test for endotoxin (Charles River Laboratories). For use in animal studies, proteins lots were required to be greater than 95% pure and have endotoxin levels less than 0.1 EU/mg.

### OMX-CV administration

After 15 minutes of alveolar hypoxia, the animals received either 200 mg/kg of OMX-CV (about 4 mL/kg by volume) as a bolus over 10 minutes, followed by continuous infusion at 70 mg/kg/hour (OMX-CV group *n* = 6), or an equivalent volume of the OMX-CV vehicle solution administered in the same manner (control group *n* = 7). At 60 minutes of alveolar hypoxia, repeat evaluation of the ESPVR was assessed by IVC occlusion.

### Physiologic monitoring

Physiologic data were continuously recorded and analyzed using the Ponemah Physiology Platform (Data Sciences International, New Brighton, MN) with Acquisition Interface, ACQ-7700 (Data Sciences International, St. Paul, MN). For calculation of total cardiac output, LPA blood flow was assumed to represent 45% of total output, as previously established in juvenile lambs by Rudolph. This was indexed to animal size by dividing by the animal’s body weight in kilograms. PVR was calculated as the difference of mean pulmonary arterial pressure and left atrial pressure divided by the indexed cardiac output. SVR was calculated as the difference of mean systemic arterial pressure and right atrial pressure divided by the indexed cardiac output. Pressure volume loop recording and analysis were performed using Labscribe software (iWorx, Dover, NH).

### Epinephrine and norepinephrine ELISA

At baseline and again at 60 minutes of hypoxia, plasma and serum samples were collected from all animals (*n* = 7 control and *n* = 6 OMX-CV) for additional analysis, including measurement of circulating catecholamines. Determination of epinephrine and norepinephrine levels in plasma was performed using a colorimetric ELISA kit (ABNOVA) according to the manufacturer’s instructions.

### Pimonidazole ELISA

In a subset of animals (*n* = 3 control and *n* = 3 OMX-CV), following the final physiologic assessment, pimonidazole (85 mg/kg) was administered intravenously over 10–15 minutes, as tolerated. Thirty minutes following the pimonidazole infusion, the animals were euthanized for tissue collection. Myocardial tissues were snap-frozen and proteins were then extracted and processed for competitive pimonidazole ELISA, as described [64]. Standard curves for the pimonidazole ELISA were fit using a five-parameter logistic equation and used to determine IC_50_ values. Values were normalized to the protein concentration in each sample and then expressed relative to the vehicle control.

### Immunohistochemistry of pimonidazole and OMX-CV

Myocardial tissues were frozen in OCT and processed for cryosectioning, followed by immunohistochemical analysis. Sections were fixed with 100% methanol for 20 minutes at −20 °C, then blocked and permeabilized with 5% BSA, 5% goat serum, and 0.1% Tween 20 for 1–2 hours at room temperature. Sections were then incubated with anti-pimonidazole (Hypoxyprobe, 1:100), anti-OMX-CV (1:200, Mouse monoclonal) antibodies overnight at 4 °C, followed by anti-rabbit or anti-mouse secondary antibodies (1:1,000, Jackson Immunoresearch Laboratories, West Grove, PA) for 2 hours at room temperature. The sections were mounted in SlowFade DAPI (Invitrogen) and imaged at the UCSF Laboratory for Cell Analysis Core with an HD AxioImager Zeiss microscope equipped with a CCD digital camera.

### Statistical analysis

Comparison of physiologic data comparing pre-hypoxic baseline to the first hypoxic physiologic time point was performed using a paired Student *t* test. Evaluation of cardiac output over the duration of the study between groups was performed using two-way ANOVA analysis. Evaluation of PVR and SVR before and after treatment between groups was performed using two-way ANOVA analysis. Pimonidazole levels were compared between groups using an unpaired Student *t* test. For ESPVR data, the slope of the ESPVR at 60 minutes of hypoxia for each ventricle of each animal was normalized to its own baseline ESPVR. These normalized values were then compared between groups using an unpaired Student *t* test. Epinephrine and norepinephrine levels at 60 minutes of hypoxia were compared between groups using unpaired Student *t* test. For all statistical tests performed, *p* ≤ 0.05 was considered to be significant. All analyses were performed using GraphPad Prism version 6.04 for Macintosh, GraphPad Software, La Jolla, CA.

## Supporting information

S1 Table Individual numerical values that underlie all numerical data, represented in either graphical or table form and arranged as individual worksheets by figure or table number. (XLSX)

## Abbreviations

bpm: beats per minute
ESPVR: end systolic pressure-volume relationship
Hb: hemoglobin
HBOC: Hb-based O_2_ carrier
H-NOX: heme-based nitric oxide/oxygen
HR: heart rate
IF: immunofluorescent
iLPA: indexed left pulmonary artery
iLPAQ: indexed left pulmonary artery flow
iLPAVR: indexed left pulmonary artery vascular resistance
iLPVR: indexed left pulmonary vascular resistance
IVC: inferior vena cava
**K**_**D**_: **dissociation constant**
LPA: left pulmonary artery
LV: left ventricle
metHb: methemoglobin
**mmHg**: **millimeters mercury**
MPA: main pulmonary artery
NO: nitric oxide
O_2_: oxygen
OMX-CV: Omniox-cardiovascular
PA: pulmonary artery
PaO_2_: arterial oxygen tension
PAP: pulmonary artery pressure
PBF: pulmonary blood flow
PEG: polyethylene glycol
PO_2_: partial pressure of oxygen
post-txt: immediately following treatment administration
pre-txt: immediately prior to treatment administration
PV: pressure-volume
PVR: pulmonary vascular resistance
RBC: red blood cell
ROS: reactive oxygen species
RV: right ventricle
SVR: systemic vascular resistance
Tt: *Thermoanaerobacter tengcongensis*
