# Correction: Preservation of myocardial contractility during acute hypoxia with OMX-CV, a novel oxygen delivery biotherapeutic

**DOI:** 10.1371/journal.pbio.3000119

**Published:** 2019-01-24

**Authors:** Jason Boehme, Natacha Le Moan, Rebecca J. Kameny, Alexandra Loucks, Michael J. Johengen, Amy L. Lesneski, Wenhui Gong, Brian D. Goudy, Tina Davis, Kevin Tanaka, Andrew Davis, Youping He, Janel Long-Boyle, Vijay Ivaturi, Jogarao V. S. Gobburu, Jonathan A. Winger, Stephen P. Cary, Sanjeev A. Datar, Jeffrey R. Fineman, Ana Krtolica, Emin Maltepe

## Notice of Republication

This article was republished on 9^th^ January, 2019 to include an author omitted in the author list of the original submission. The author contributions have been amended to reflect the additional author, Brian D. Goudy.

The amended author list is as follows:

Jason Boehme^1‡^, Natacha Le Moan^2‡^, Rebecca J. Kameny^1^, Alexandra Loucks^2^, Michael J. Johengen^1^, Amy L. Lesneski^1^, Wenhui Gong^1^, Brian D. Goudy^1^, Tina Davis^2^, Kevin Tanaka^2^, Andrew Davis^2^, Youping He^1^, Janel Long-Boyle^3,4^, Vijay Ivaturi^4,5^, Jogarao V. S. Gobburu^4,5^, Jonathan A. Winger^2^, Stephen P. Cary^2^, Sanjeev A. Datar^1^, Jeffrey R. Fineman^1,4^, Ana Krtolica^2^*, Emin Maltepe^1,4^*

**1** Department of Pediatrics, University of California, San Francisco, San Francisco, California, United States of America, **2** Omniox, Inc., San Carlos, California, United States of America, **3** Department of Clinical Pharmacology, University of California, San Francisco, San Francisco, California, United States of America, **4** Initiative for Pediatric Drug and Device Development (iPD3), San Francisco, California, United States of America, **5** School of Pharmacy, University of Maryland, Baltimore, United States of America

^‡^ These authors share first authorship on this work.

* akrtolica@omnioxinc.com (AK); emin.maltepe@ucsf.edu (EM)

Please download this article again to view the correct version.

## Author Contributions

**Conceptualization**: Jonathan A. Winger, Stephen P. Cary, Jeffrey R. Fineman, Ana Krtolica, Emin Maltepe.

**Data curation**: Jason Boehme, Natacha Le Moan, Rebecca J. Kameny, Brian D. Goudy, Janel Long-Boyle, Vijay Ivaturi, Jogarao V. S. Gobburu, Jonathan A. Winger, Sanjeev A. Datar, Jeffrey R. Fineman, Ana Krtolica, Emin Maltepe.

**Formal analysis**: Jason Boehme, Natacha Le Moan, Rebecca J. Kameny, Janel Long-Boyle, Vijay Ivaturi, Sanjeev A. Datar, Jeffrey R. Fineman, Ana Krtolica, Emin Maltepe.

**Funding acquisition**: Stephen P. Cary, Jeffrey R. Fineman, Ana Krtolica, Emin Maltepe.

**Investigation**: Jason Boehme, Rebecca J. Kameny, Alexandra Loucks, Michael J. Johengen, Amy L. Lesneski, Wenhui Gong, Brian D. Goudy, Tina Davis, Andrew Davis, Youping He, Vijay Ivaturi, Jogarao V. S. Gobburu, Sanjeev A. Datar, Jeffrey R. Fineman, Ana Krtolica, Emin Maltepe.

**Methodology**: Jason Boehme, Rebecca J. Kameny, Alexandra Loucks, Michael J. Johengen, Amy L. Lesneski, Wenhui Gong, Brian D. Goudy, Tina Davis, Kevin Tanaka, Andrew Davis, Youping He, Janel Long-Boyle, Vijay Ivaturi, Jonathan A. Winger, Sanjeev A. Datar, Jeffrey R. Fineman, Ana Krtolica, Emin Maltepe.

**Project administration**: Natacha Le Moan, Rebecca J. Kameny, Alexandra Loucks, Jeffrey R. Fineman, Ana Krtolica, Emin Maltepe.

**Resources**: Natacha Le Moan, Rebecca J. Kameny, Alexandra Loucks, Jeffrey R. Fineman, Ana Krtolica, Emin Maltepe.

**Supervision**: Natacha Le Moan, Rebecca J. Kameny, Michael J. Johengen, Kevin Tanaka, Youping He, Janel Long-Boyle, Vijay Ivaturi, Jogarao V. S. Gobburu, Jonathan A. Winger, Stephen P. Cary, Sanjeev A. Datar, Jeffrey R. Fineman, Ana Krtolica, Emin Maltepe.

**Validation**: Jason Boehme, Natacha Le Moan, Rebecca J. Kameny, Brian D. Goudy, Janel Long-Boyle, Sanjeev A. Datar, Emin Maltepe.

**Visualization**: Jason Boehme, Natacha Le Moan, Rebecca J. Kameny, Alexandra Loucks, Ana Krtolica, Emin Maltepe.

**Writing – original draft**: Jason Boehme, Jeffrey R. Fineman, Emin Maltepe.

**Writing – review & editing**: Jason Boehme, Natacha Le Moan, Alexandra Loucks, Janel LongBoyle, Jonathan A. Winger, Stephen P. Cary, Sanjeev A. Datar, Jeffrey R. Fineman, Ana Krtolica, Emin Maltepe.

## References

[1] Boehme J, Le Moan N, Kameny RJ, Loucks A, Johengen MJ, Lesneski AL, et al (2018) Preservation of myocardial contractility during acute hypoxia with OMX-CV, a novel oxygen delivery biotherapeutic. PLoS Biol 16(10): e2005924 10.1371/journal.pbio.2005924 30335746 PMC6193608

